# MoCbp7, a Novel Calcineurin B Subunit-Binding Protein, Is Involved in the Calcium Signaling Pathway and Regulates Fungal Development, Virulence, and ER Homeostasis in *Magnaporthe oryzae*

**DOI:** 10.3390/ijms24119297

**Published:** 2023-05-26

**Authors:** Zi-He Wang, Zi-Fang Shen, Jing-Yi Wang, Ying-Ying Cai, Lin Li, Jian Liao, Jian-Ping Lu, Xue-Ming Zhu, Fu-Cheng Lin, Xiao-Hong Liu

**Affiliations:** 1 State Key Laboratory of Rice Biology and Breeding, Institute of Biotechnology, Zhejiang University, Hangzhou 310058, China; 2 State Key Laboratory for Managing Biotic and Chemical Treats to the Quality and Safety of Agro-Products, Institute of Plant Protection and Microbiology, Zhejiang Academy of Agricultural Sciences, Hangzhou 310021, China; 3 College of Life Sciences, Zhejiang University, Hangzhou 310058, China

**Keywords:** *Magnaporthe oryzae*, calcium signaling, calcineurin, virulence, endoplasmic reticulum stress

## Abstract

Calcineurin, a key regulator of the calcium signaling pathway, is involved in calcium signal transduction and calcium ion homeostasis. *Magnaporthe oryzae* is a devastating filamentous phytopathogenic fungus in rice, yet little is known about the function of the calcium signaling system. Here, we identified a novel calcineurin regulatory-subunit-binding protein, MoCbp7, which is highly conserved in filamentous fungi and was found to localize in the cytoplasm. Phenotypic analysis of the *MoCBP7* gene deletion mutant (Δ*Mocbp7*) showed that MoCbp7 influenced the growth, conidiation, appressorium formation, invasive growth, and virulence of *M. oryzae*. Some calcium-signaling-related genes, such as *YVC1*, *VCX1*, and *RCN1*, are expressed in a calcineurin/MoCbp7-dependent manner. Furthermore, MoCbp7 synergizes with calcineurin to regulate endoplasmic reticulum homeostasis. Our research indicated that *M. oryzae* may have evolved a new calcium signaling regulatory network to adapt to its environment compared to the fungal model organism *Saccharomyces cerevisiae*.

## 1. Introduction

Calcium ions are widely present in cells as universal signaling mediators, and they affect a series of biological processes [1]. In fungi, as in other higher organisms, the resting cytosol shows a very low free calcium ion concentration (between 50 nM and 200 nM). When cells are stimulated by environmental cues or developmental factors, calcium ions from external stores or organelles (such as endoplasmic reticulum (ER) and vesicles) enter the cytosol along calcium channels and initiate downstream signaling by changing the conformation of calcium-binding proteins [2,3,4]. The free calcium ion level in the cytosol usually shows a rapid and transient increase after stimulation. It eventually shows an exponential decline, which depends on the inverse concentration gradient transport by calcium transport proteins located at the plasma and intracellular membranes and is important for intracellular calcium ion homeostasis [5].

Calcineurin (CN) is a calcium ion/calmodulin (CAM)-binding protein that is conserved in eukaryotes [6]. As a serine/threonine protein phosphatase, it consists of a catalytic subunit (calcineurin A, CNA) and a regulatory subunit (calcineurin B, CNB). The catalytic subunit contains a central calmodulin-binding region and an N-terminal phosphatase domain, while the regulatory subunit possesses four EF-hand motifs that bind calcium ions with high affinity [7]. When the calcium ion concentration in the cytosol is transient, calcineurin relies on calcium ions or the calcium sensor protein, calmodulin, to dephosphorylate downstream targets, such as a critical transcription factor, Crz1, which is a homolog of the nuclear factor of activated T cells (NFAT) in mammals and is involved in calcium signaling transduction and calcium ion homeostasis in fungi [8,9]. Some endogenous regulators have been identified to regulate calcineurin activity, such as the calcineurin temperature suppressor, Cts1 [10], and the calcineurin regulator, Rcn1 [11], found in fungi. In addition, some immunosuppressive drugs, such as cyclosporin A and FK506, can effectively inhibit calcineurin in the presence of their respective cytoplasmic immunophilin, cyclophilin, and FK506-binding protein [7].

The ER is a complex organelle responsible for many biological processes such as protein synthesis and processing, lipid metabolism, and calcium ion storage in fungi [12]. A higher concentration of calcium ions is essential for enzyme activity. In *S. cerevisiae*, the steady-state calcium ion concentration in the ER is approximately 100 times higher than in the cytosol [13]. If calcium homeostasis in the ER is dysregulated, ER stress will be induced, the unfolded protein response (UPR) pathway, which drives adaptive protein folding, is triggered to keep the ER in a steady state. [12,14]. The activation of the UPR pathway also induces endoplasmic reticulum autophagy (ER-phagy), which reshapes ER homeostasis by degrading damaged and redundant parts of the ER [15,16]. Numerous studies have reported the role of calcineurin, calcium transport proteins, and other calcium-signaling-related proteins in response to ER stress [14,17,18,19,20], suggesting that the calcium signaling pathway is necessary for ER homeostasis in fungi.

*Magnaporthe oryzae* poses a serious threat to rice production worldwide and has been receiving widespread attention [21,22]. Asexual conidia of *M. oryzae* can germinate on rice leaves under suitable environmental conditions. The conidia germinate and form polarized germ tubes, the tips of which swell to form a specialized infection structure termed appressorium. The appressorium can be fixed on the leaf surface and generate up to 8.0 MPa of turgor pressure by accumulating organic molecules such as glycerol. The turgor pressure will eventually be converted into a mechanical force to form a penetration peg at the base of the appressorium to breach the host cuticle, and the invasive hyphae will expand within the rice tissue to cause disease [23,24]. Typical disease spots form on the leaf surface, and the mature conidia can start a new round of spread with the help of wind and dew after 5–7 days [25,26]. In the last two decades, many conserved signaling pathways have been shown to be involved in virulence-related signaling and in response to external physical and chemical signals in *M. oryzae,* such as the cAMP-protein kinase A (cAMP-PKA) pathway and the Pmk1 mitogen-activated protein kinase (Pmk1-MAPK) pathway [27,28,29,30]; the production of turgor pressure depends on autophagy to degrade conidial contents [24,31]; the Mps1-MAPK pathway regulates the growth of invasive hyphae and inhibits the host defense response [32,33]; and the target of rapamycin (TOR) signaling pathway, which regulates autophagy and is necessary for appressorial development and invasive growth [34,35].

However, the underlying regulatory mechanisms of the calcium signaling pathway remain largely unknown in *M. oryzae*. Many calcium-signal-related proteins are involved in the regulation of *M. oryzae* virulence, including genes encoding calcineurin, calcineurin A subunit (MoCna1), and calcineurin B subunit (MoCnb1). MoCnb1 is especially important in regulating the virulence of *M. oryzae* [36]. Here, we reported a novel gene, MGG_16441, which encodes a protein that was shown to interact with MoCnb1; we named it MoCbp7 (calcineurin-binding protein). MoCbp7 was localized in the cytoplasm and participated in the calcium signaling pathway, was involved in calcium ion homeostasis and ER homeostasis, and was found to be essential for growth, conidiation, appressorium formation, and virulence.

## 2. Results

### 2.1. Identification of MoCbp7

**Figure 1:**
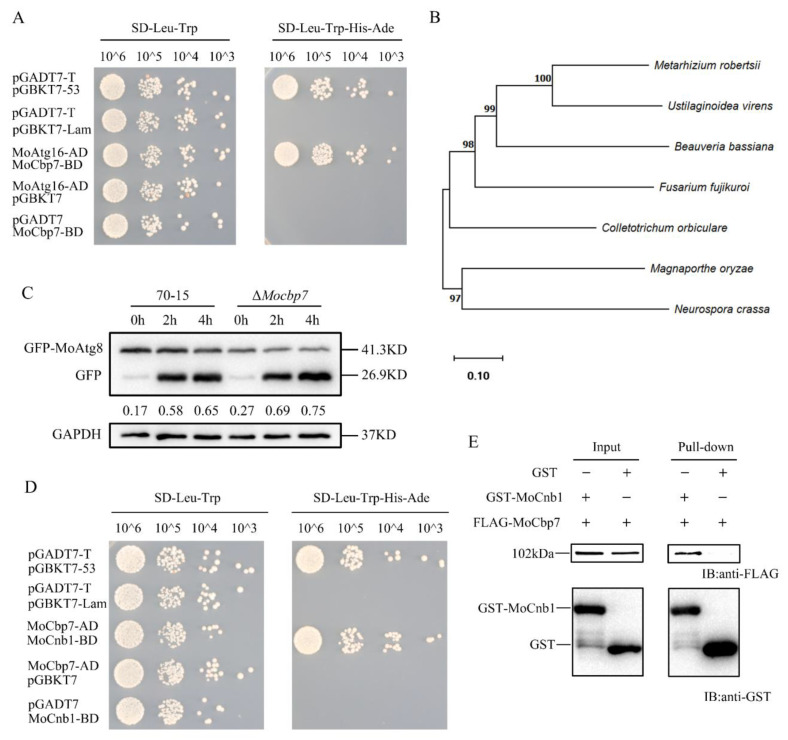
Identification of MoCbp7. (**A**) Yeast two-hybrid assay of MoCbp7 and MoAtg16 interaction. (**B**) Phylogenetic analysis of MoCbp7 and homologues of MoCbp7 in several filamentous fungi. MEGA11 was used to construct the phylogenetic tree using the neighbor-joining method with 1000 bootstrap replicates and the Poisson model. The filamentous fungi can be found in GeneBank with the following accession numbers: XP_003710568.1 (*M. oryzae*); XP_008601432.1 (*Beauveria bassiana*); XP_023433455.1 (*Fusarium fujikuroi*); XP _007819772.2 (*Metarhizium robertsii*); XP_956171.2 (*Neurospora crassa*); TDZ23940.1 (*Colletotrichum orbiculare*); and XP_042999839.1(Ustilaginoidea virens). (**C**) Autophagy levels of the wild-type 70-15 and Δ*Mocbp7* in liquid CM and SD-N induction were detected by examining the degradation level of GFP-MoAtg8. (**D**) Yeast two-hybrid assay for the interaction of MoCbp7 and MoCnb1. pGBKT7-53 and pGADT7-T were used as positive controls. pGBKT7-Lam and pGADT7-T were used as negative controls. (**E**) The pulldown assay for the detection of the interaction of MoCbp7 and MoCnb1.

The process of autophagy-mediated infection of plants through appressorial turgor generation has been extensively studied [24]. The autophagy process depends on the regulation of autophagy-related genes (Atg), which enables the orderly clearance and recycling of intracellular waste. Atg16 is a core protein of autophagy and is essential for autophagosome formation [37]. Using MoAtg16 as bait, a novel calcineurin regulatory-subunit-binding gene (MGG_16441) was identified in the screening of a cDNA library of rice blast fungus, and the interaction was further verified with a yeast two-hybrid assay (Figure 1A). Blastp (http://blast.ncbi.nlm.nih.gov, accessed on 5 October 2022) showed that MGG_16441 (designated as MoCbp7) is conserved in filamentous fungi by a phylogenetic analysis (Figure 1B). However, their biological functions have not been characterized in any other species. Thus, we further investigated its role in *M. oryzae*.

In order to verify the functions of MoCbp7, we obtained a null mutant by a high-throughput knockout system and generated a complemented strain containing a 3 × FLAG tag. To ascertain the effect of MoCbp7 on autophagy, the autophagy flux was detected by the degradation rate of GFP-MoAtg8, an autophagy marker, in Δ*Mocbp7* and wild-type 70-15. Western blot analysis revealed that the autophagy flux is slightly accelerated in Δ*Mocbp7* (Figure 1C). This implied that MoCbp7 may not be directly involved in autophagy-mediated infection. Then, using MoCbp7 as bait, we performed a screening of the rice blast fungus cDNA library again, and the potential interacting proteins are listed in Table S1. Interestingly, a calcineurin B subunit (MoCnb1), which was previously reported to be knockout lethal and to act as a key protein in infection mediation in *M*. *oryzae* [36], was found to interact with MoCbp7 in a further yeast two-hybrid validation (Figure 1D) and pulldown assay (Figure 1E). Hence, we supposed that MoCbp7 interacts with MoCnb1 to regulate the calcineurin-mediated calcium signaling pathway.

### 2.2. MoCbp7 Is Localized in the Cytoplasm of M. oryzae

**Figure 2:**
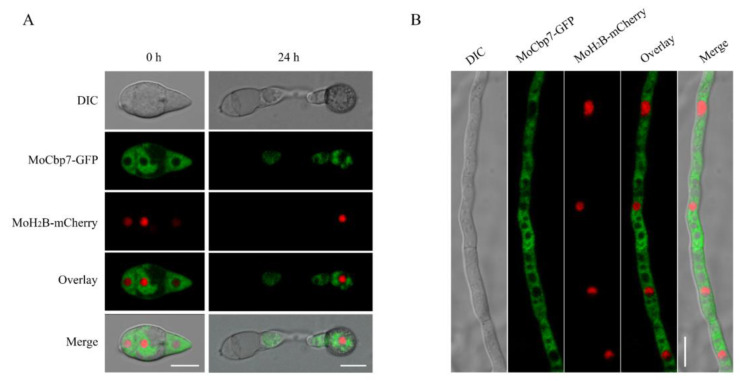
MoCbp7 is localized in the cytoplasm of *M. oryzae*. (**A**) Fluorescence signals of MoCbp7-GFP and MoH_2_B-mCherry at conidial and appressorial stages; bar, 10 μm. (**B**) Fluorescence signals of MoCbp7-GFP and MoH_2_B-mCherry at mycelial stage; bar, 10 μm.

According to previous reports, the cytoplasm is where calcineurin is mainly localized [11]. To characterize the subcellular localization of MoCbp7, the MoCbp7-GFP fusion protein and a nuclear location marker, MoH_2_B-mCherry, were co-expressed in the Δ*Mocbp7* mutant. The fluorescent signal of MoCbp7-GFP appeared to avoid the nucleus-like structures and dispersed in the cytoplasm during the conidia and hyphae stages as well as the appressorium stage at 24 h post-inoculation (hpi) (Figure 2A,B). However, the fluorescent signal of MoCbp7-GFP spread throughout the cytoplasm in the conidia and hyphae stages, while the fluorescent signal scattered as a punctate pattern throughout the appressorium stage at 24 hpi (Figure 2A,B).

### 2.3. MoCbp7 Is Involved in the Calcium Signaling Pathway

**Figure 3:**
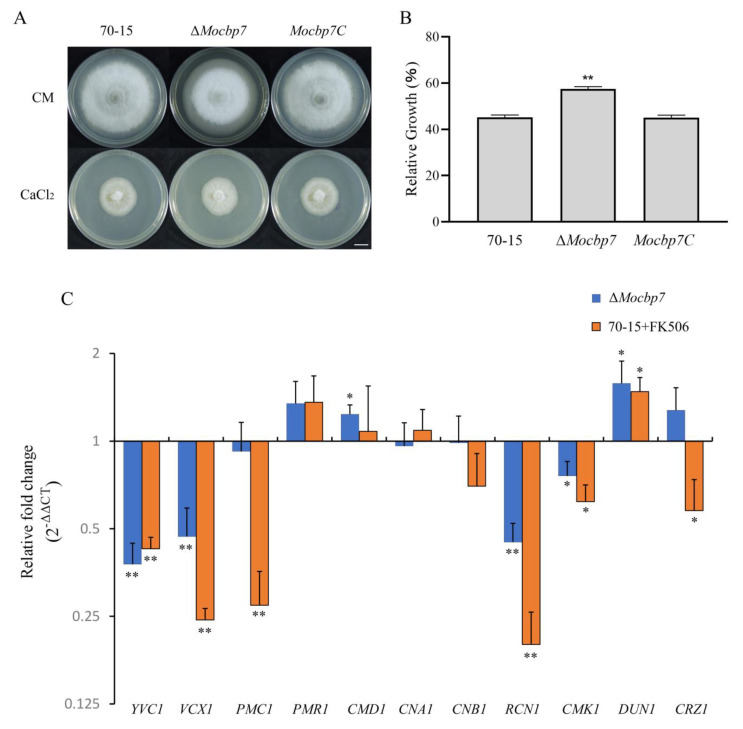
MoCbp7 is involved in the calcium signaling pathway. (**A**) Wild-type 70-15, Δ*Mocbp7,* and *Mocbp7C* were inoculated on CM plates containing 0.3 M CaCl_2_ at 25 °C with 16 h photoperiod for 9 days; bar, 1 cm. (**B**) Relative growth rates of wild-type 70-15, Δ*Mocbp7*, and *Mocbp7C* on CM plates containing 0.3 M CaCl_2_. Statistical differences were analyzed by GraphPad Prism 8.0 via Tukey’s test (** p < 0.01). (**C**) Relative transcriptional expression of calcium-signaling-related genes in Δ*Mocbp7* and wild-type 70-15 treated with 10 μg/mL of FK506 for 1 h. Statistical differences were analyzed by GraphPad Prism 8.0 via Tukey’s test (* *p* < 0.05, ** *p* < 0.01).

The calcineurin B subunit was previously reported to participate in the calcium signaling pathway [6,38]. Thus, a follow-up investigation was conducted to determine whether MoCbp7 is also involved in the calcium signaling pathway in light of the interaction between MoCbp7 and MoCnb1. The mycelium plugs of wild-type 70-15, Δ*Mocbp7*, and *Mocbp7C* were inoculated on CM plates containing 0.3 M CaCl_2_, and the relative growth rate of the Δ*Mocbp7* mutant was significantly higher than that of wild-type 70-15 and *Mocbp7C* strains after 9 days post-inoculation (dpi) (Figure 3A,B), which suggested that the mechanism for Δ*Mocbp7* to respond to the high-external-calcium environment may been disturbed.

Next, the expression levels of genes associated with the calcium signaling pathway, including calcium transport proteins (*YVC1*, *VCX1, PMC1,* and *PMR1* [36]), calcium signaling sensors (*CMD1, CNA1, CNB1, CMK1,* and *DUN1* [36]), and calcineurin-binding proteins (*RCN2* [11] and *CRZ1* [9]), were further analyzed by quantitative real-time PCR (qRT-PCR) in both the wild-type 70-15 treated with a calcineurin inhibitor (FK506) and the Δ*Mocbp7* mutant. As shown in Figure 3C, the expression levels of *YVC1*, *VCX1*, and *RCN1* were significantly downregulated in the Δ*Mocbp7* mutant, similar to those of the wild-type 70-15 treated with FK506. However, the expression level of the transcription factor *CRZ1* and its possible target, *PMC1*, were markedly reduced in the wild type treated with FK506, while there was a slight effect in the Δ*Mocbp7* mutant. Additionally, both in the treated wild-type 70-15 and the Δ*Mocbp7* mutant, there were no appreciable changes (within a 0.5-fold change) in the expression level of several key genes, including *PMR1*, *CMD1*, *CNA1*, and *CNB1*, indicating that the expression level of these genes may not be considerably impacted by the suppression of calcineurin activity or the deletion of *MoCBP7*. In conclusion, many important calcium-signaling-related proteins function in a calcineurin-dependent manner, and MoCbp7 exhibited functional synergy as a calcineurin-binding protein, suggesting that MoCbp7 was involved in the calcium signaling pathway and was essential for maintaining intracellular calcium homeostasis. However, MoCbp7 does not appear to be involved in negatively regulating calcineurin/CRZ1 signaling in *M. oryzae*.

### 2.4. MoCbp7 Is Essential for Asexual Growth, Conidiation, and Appressorium Formation

**Figure 4:**
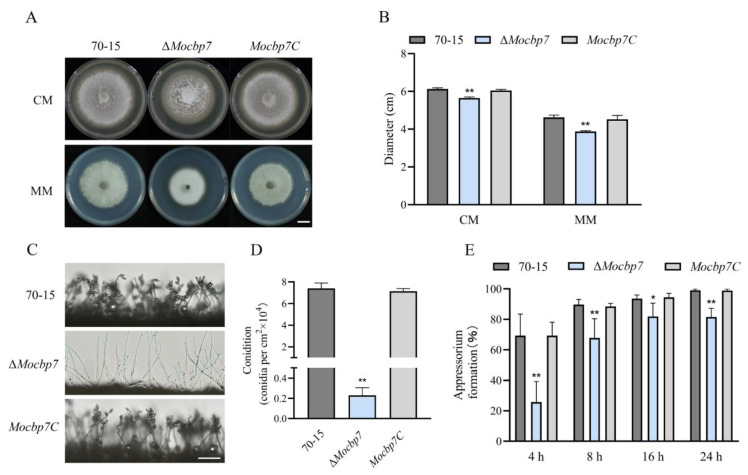
MoCbp7 is essential for asexual growth, sporulation, and appressorium formation. (**A**) Colony morphology of wild-type 70-15, Δ*Mocbp7,* and *Mocbp7C* was photographed after 9 dpi at 25 °C; bar, 1 cm. (**B**) Colony diameters of wild-type 70-15, Δ*Mocbp7*, and *Mocbp7C*. (**C**) Conidiophore morphology of wild-type 70-15, Δ*Mocbp7,* and *Mocbp7C* after 24 hpi at 28 °C; bar, 100 μm. (**D**) Conidia numbers of 70-15, Δ*Mocbp7*, and *Mocbp7C* after 9 dpi. (**E**) Appressorium formation rates of wild-type 70-15, Δ*Mocbp7*, and *Mocbp7C*. The appressoria were induced on hydrophobic coverslips for 4 h, 8 h, 16 h, and 24 h. Statistical differences were analyzed by GraphPad Prism 8.0 via Tukey’s test (* *p* < 0.05, ** *p* < 0.01).

To more comprehensively investigate the function of MoCbp7, we conducted a systematic investigation of the Δ*Mocbp7* mutant in terms of colony growth, conidiation, and appressorium formation in *M. oryzae*. As shown in Figure 4A, mycelium turned white when *MoCBP7* was deleted, and the colony growth rate of the Δ*Mocbp7* mutant was slower compared to wild-type 70-15 and *Mocbp7C* (Figure 4A,B) both in complete medium (CM) and minimal medium (MM). Further investigation of the conidiation ability revealed that, in contrast to the wild-type 70-15 and *Mocbp7C*, which possessed the normal clustering conidia, the Δ*Mocbp7* mutant showed far fewer conidia and had longer and unbranched conidiophores (Figure 4C). As shown in Figure S2 and Figure 4D, the Δ*Mocbp7* mutant displayed a severe conidiation deficiency but a normal conidial shape. Plant infection in *M. oryzae* is significantly influenced by the appressorium, a typical pathogenic fungal infection structure [24]. Therefore, we further observed the morphology of the developing appressorium in the Δ*Mocbp7* mutant. Similar to the wild-type 70-15 and *Mocbp7C*, the conidia germinated normally in the Δ*Mocbp7* mutant. However, appressorium formation proceeded at a slower rate, with about 18.48% of conidia still unable to form an appressorium at 24 hpi (Figure 4E and Figure S2). In conclusion, our phenotypic analysis indicated that MoCbp7 is essential for asexual growth, conidiation, and appressorium formation in *M. oryzae*.

### 2.5. MoCbp7 Is Required for Virulence

**Figure 5:**
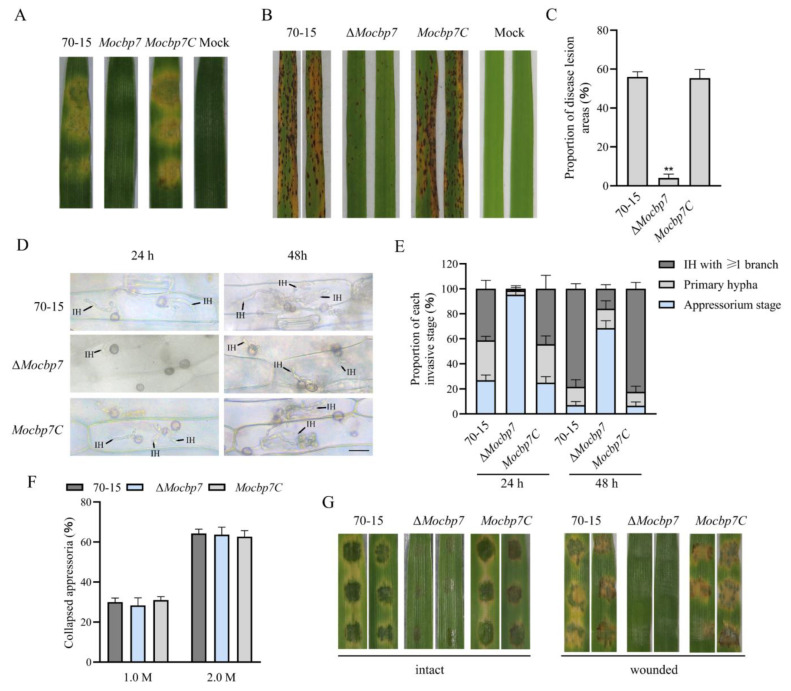
MoCbp7 is required for full pathogenicity. (**A**) The mycelium plugs of wild-type 70-15, the Δ*Mocbp7* mutant, and *Mocbp7C* were inoculated on detached barley leaves after 4 dpi. (**B**) Disease symptoms on rice seedlings (CO-39) sprayed with conidial suspensions and photographed after 6 dpi. (**C**) The area of the diseased spot was calculated via Adobe Photoshop 2022 (Version 23.0) after 7 dpi. Statistical differences were analyzed by GraphPad Prism 8.0 via Tukey’s test (** p < 0.01). (**D**) The appressorial infection of 70-15, the Δ*Mocbp7* mutant, and *Mocbp7C*. The conidial suspensions (5 × 10^4^ conidia/mL) were inoculated on detached barley leaves. Then the barley leaves were decolorized with methanol and observed by a light microscope after 24 hpi and 48 hpi; bar, 20 μm. (**E**) Statistical analysis of the proportions of wild-type 70-15, ΔMocbp7 mutant, and Mocbp7C in different invasion stages at 24 hpi and 48 hpi. Experiments were performed in triplicate with 100 appressoria each time. (**F**) Collapse rates of mature appressoria in wild-type 70-15, the Δ*Mocbp7* mutant, and *Mocbp7C* after glycerol treatment. Experiments were performed in triplicate with 100 appressoria each time. Statistical differences were analyzed by GraphPad Prism 8.0 via Tukey’s test. (**G**) Disease symptoms on intact detached barley leaves and wounded detached barley leaves with conidial suspensions (5 × 10^4^ conidia/mL) from wild-type 70-15, Δ*Mocbp7*, and *Mocbp7C* photographed after 3 dpi.

To determine the effect of MoCbp7 on the virulence of *M. oryzae*, as shown in Figure 5A, the mycelium plugs of wild-type 70-15, Δ*Mocbp7*, and *Mocbp7C* were inoculated on detached barley leaves and photographed after 4 dpi. In contrast to the barley leaves inoculated with wild-type 70-15 and *Mocbp7C*, which showed typical severe disease symptoms, the barley leaves inoculated with the Δ*Mocbp7* mutant showed little visible damage. Then, conidial suspensions (5 × 10^4^ conidia/mL) were acquired for the subsequent spray infection assays in order to further validate whether the defect in virulence of the Δ*Mocbp7* mutant was exclusively caused by the defect in conidiation. Similarly, in the spray infection assays with 2-week-old rice seedlings (CO-39), numerous typical spindle-shaped lesions were observed on leaves sprayed with the conidia of wild-type 70-15 or the complementary strain *Mocbp7C*, but only slightly limited necrosis was observed in the Δ*Mocbp7* mutant after 6 dpi (Figure 5B,C). Next, the invasive stage of the appressorium was classified after observation of the infection of detached barley leaves under an electron microscope (Figure 5D,E). At 24 hpi, Δ*Mocbp7* mutant barely developed invasive hypha (IH), and less than 5% of the appressoria had developed penetration pegs. In contrast, 72.67% of the appressoria in the wild-type 70-15 produced IH, of which 41.25% had one or more branches. At 48 hpi, nearly 95% of the appressoria in wild-type 70-15 and *Mocbp7C* had penetrated the host cuticle and produced IH with multiple branches that spread to adjacent cells. This is compared to 68.56% of the Δ*Mocbp7* mutant appressoria, which were still in the non-infected stage, and only 16.33% of the IH could form branches but did not develop to adjacent cells, which indicated the severely impaired invasion capacity of appressoria in the Δ*Mocbp7* mutant.

Previous studies have shown that a critical component of the penetrating pegs’ ability to penetrate the host cuticle and achieve invasion is the enormous turgor pressure in the appressorium [24]. To investigate whether the defective appressorial infection of the Δ*Mocbp7* mutant is due to insufficient turgor pressure accumulated in the appressoria, 1.0 M and 2.0 M concentrations of exogenous glycerol were applied to the appressorium, and the results for the Δ*Mocbp7* mutant were not significantly different than those of 70-15 and *Mocbp7C,* as shown in Figure 5F. Additionally, the Δ*Mocbp7* mutant showed a consistent result in both intact and wounded (cuticle-damaged) barley leaves, indicating that the deficiency in conidial virulence was not due to the insufficient appressoria turgor. In summary, we concluded that *MoCBP7* is a pathogenicity-related gene that is required for appressorial penetration and invasive growth.

### 2.6. MoCbp7 Cooperates with Calcineurin to Regulate ER Homeostasis in M. oryzae

**Figure 6:**
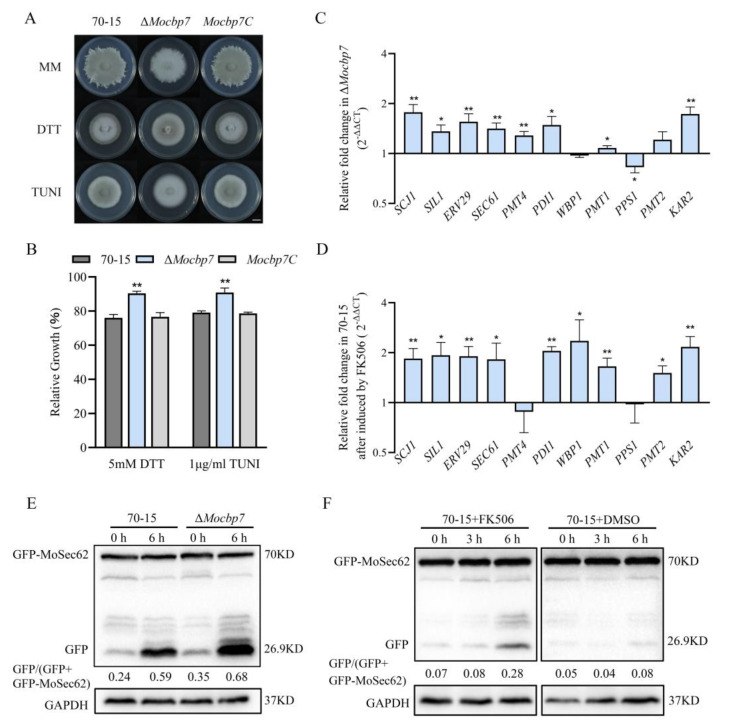
MoCbp7 cooperates with calcineurin to regulate ER homeostasis in *M. oryzae*. (**A**) Wild-type 70-15, Δ*Mocbp7*, and *Mocbp7C* were cultured in the dark for 9 days in MM medium supplemented with 5 mM DTT or 1 μM TUNI; bar, 1 cm. (**B**) Relative growth rates of wild-type 70-15, Δ*Mocbp7*, and *Mocbp7C* under DTT and TUNI stress. (**C**) Relative expression of UPR-pathway-related genes in the Δ*Mocbp7* mutant compared with wild-type 70-15 after incubation in CM medium. (**D**) Relative expression of UPR-pathway-related genes in wild-type 70-15 after 10 μg/mL FK506 treatment for 1 h. Statistical differences were analyzed by GraphPad Prism 8.0 via Tukey’s test (* *p* < 0.05, ** *p* < 0.01). (**E**) The ER-phagy levels of wild-type 70-15 and Δ*Mocbp7* were detected by western blot in liquid CM or 6 h of 5 mM DTT treatment. (**F**) ER-phagy level of wild-type 70-15 in liquid CM and after treatment with FK506 was detected. In order to exclude the influence of the FK506 solvent on the determination results, dimethyl sulfoxide (DMSO) was used in wild-type 70-15. GAPDH was used as a control.

The ER, one of the major organelles for storing calcium ions, is subject to precise concentration regulation [39]. Numerous studies have shown that the calcium signaling pathway participates in ER stress adaptation, whereas a reduction in the calcium content in the ER induces ER stress [12]. To better comprehend the relationship between the calcium signaling system and ER, two ER stress inducers, dithiothreitol (DTT) and tunicamycin (TUNI), were added to the MM media to test the sensitivity of the Δ*Mocbp*7 mutant. In MM media supplemented with 5 mM DTT or 1 g/mL TUNI, the relative growth rate of the Δ*Mocbp*7 mutant was higher than that of wild-type 70-15 and Mocbp7C. (Figure 6A,B).

To further investigate ER homeostasis in the Δ*Mocbp7* mutant, expression of the relevant genes involved in the UPR pathway was analyzed by qRT-PCR [40]. Interestingly, the expression of UPR-pathway-related genes was generally increased by 0.5 to 1-fold in the Δ*Mocbp7* mutant under CM culture conditions compared with the wild-type 70-15 (Figure 6C). Similar results were attained when the calcineurin inhibitor tacrolimus (FK506) was added to the wild type (Figure 6D).

The ER stress response initially activates the UPR pathway. The apoptotic process is triggered by the UPR when ER homeostasis is disturbed, and thus, ER-phagy is initiated [41]. To further demonstrate the role of MoCbp7 in ER homeostasis, a marker of ER-phagy, GFP-MoSec62, was transferred to the wild-type 70-15 and Δ*Mocbp7*, and the level of ER-phagy was determined by monitoring the degradation level of the expressed fusion protein. The ER-phagy level was higher in the Δ*Mocbp7* mutant when cultured in liquid CM (Figure 6E). After treatment with 5 mM DTT for 6 h, the level of ER degradation was still significantly higher in the Δ*Mocbp7* mutant than in the wild type (Figure 6E). Similar results were found in the FK506-treated wild type (Figure 6F). The spontaneous activation of the UPR pathway and ER-phagy in Δ*Mocbp7* implies that MoCbp7 is involved in the regulation of ER homeostasis and that regulation may be carried out in cooperation with calcineurin in *M. oryzae*. Furthermore, our data supported previous studies that suggest that the inhibition of calcineurin activity causes ER stress, which in turn leads to the activation of the UPR pathway and ER-phagy, suggesting that calcineurin is a regulator of ER homeostasis in *M. oryzae*.

## 3. Discussion

Calcium signaling is affected by the flux of calcium ions from external and internal stores. In the calcium signaling system, calcineurin function as a regulatory hub, delivering calcium signaling information and regulating calcium ion homeostasis so that intracellular calcium signaling is ordered in a wave or transient form [1,5]. The lifestyle requirements for a pathogen have shaped specific signaling modules in these organisms, and unlike higher eukaryotes, filamentous fungi have distinct calcium signaling networks, showing peculiarities in the purposes and specific traits of calcium signaling pathways [5,42]. Here we identified a specific calcineurin B subunit-binding protein, MoCbp7, which was found to play pleiotropic roles development, conidiation, appressorium formation, invasive growth, and virulence in *M. oryzae* (Figure 4 and Figure 5). Additionally, we found that MoCbp7 and calcineurin cooperated to maintain endoplasmic reticulum homeostasis, revealing a novel mechanism for calcium signaling and endoplasmic-reticulum-dependent cell homeostasis and providing new avenues for drug development.

Calcineurin-mediated calcium signaling plays an important role in growth, conidiation, and virulence [43,44] as well as various environmental stress responses, such as high temperature, high pH, and ER stress [45]. However, the precise mechanism of how calcineurin is involved in numerous signaling processes is still uncertain. Comparative genomic analysis revealed that a greater number of calcium-signaling-related proteins are present in *N. crassa* and *M. oryzae* compared to *S. cerevisiae*, and filamentous fungi have more redundant Ca^2+^-permeable channels, cation/proton exchangers, and P-ATPases, which is likely due to their more complicated cellular organization and filamentous growth form. This implies that filamentous fungi have a more complex calcium signaling response mechanism than *S. cerevisiae* [4]. Recently, new studies of various calcineurin-mediated calcium signals in fungi have emerged. However, only one calcineurin downstream transcription factor, Crz1, has been identified in fungi, which can directly interact with the calcineurin A subunit and initiate nuclear localization to mediate downstream gene transcription [9,46]. A total of 140 of the 346 MoCrz1-binding genes discovered in *M. oryzae* are targets regulated in a calcineurin/Crz1-dependent manner [47]. Additionally, more than 30 calcium/Rcn1-dependent genes were discovered to be direct targets of MoCrz1, in which Rcn1 is a regulator of calcineurin [11]. However, the underlying regulatory mechanisms of calcineurin remain largely unknown in *M. oryzae* as well as in many others. Here we identified a novel calcineurin regulatory-subunit-binding protein, MoCbp7, which is crucial for the calcium signaling pathway in *M. oryzae*. Since MoCbp7 is only found in filamentous fungi, we speculated that it may be a novel protein evolved only in filamentous fungi that enables them to survive in their particular environment.

**Figure 7:**
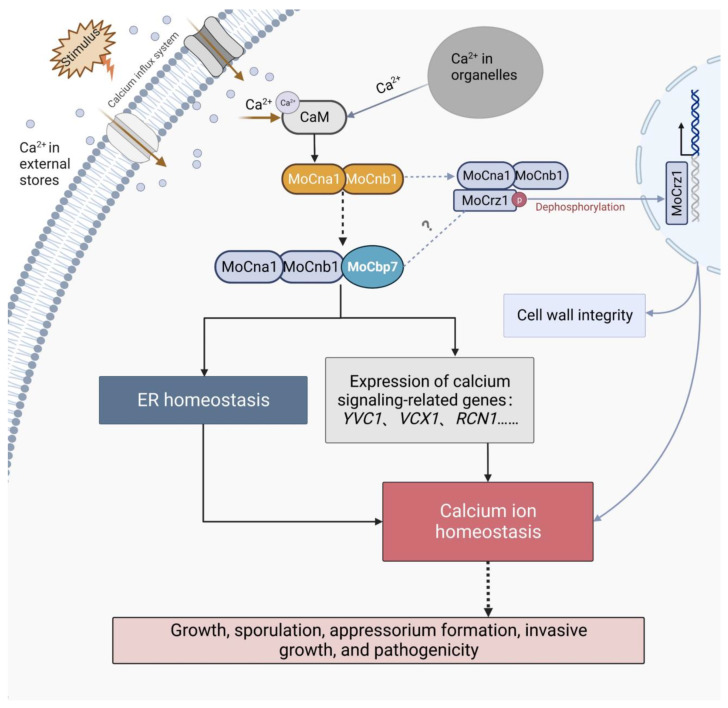
Model of the involvement of MoCbp7 in the calcineurin-mediated calcium signaling pathway. The calcium ion flux from external and internal stores initiates calcium signaling. Calcineurin acts as a regulatory hub in the calcium signaling pathway. Both MoCbp7 and MoCrz1 are important participants in the calcineurin-mediated calcium signaling pathway. They may precisely regulate the calcium signaling at different temporal and spatial levels to maintain intracellular calcium ion homeostasis. The correct transmission of calcium ion signals, that is, calcium ion homeostasis, is crucial for fungal development and virulence in *M. oryzae*. Created with BioRender.com.

Calcineurin plays an important role in ER stress. In *S*. *cerevisiae*, calcium uptake activates calcineurin activity to protect cells from damage by prolonged ER stress, but large amounts of calcium ions trigger programmed cell death [48,49]. Our experiments demonstrated that in *M. oryzae*, both FK506 treatment and deletion of MoCbp7 caused ER stress, triggering constitutive activation of the UPR pathway and ER-phagy (Figure 6), which indicates that ER homeostasis is regulated by calcineurin. ER homeostasis is involved in the pathogenicity of pathogens. According to previous reports, activation of the UPR pathway or stimulation of ER-phagy affect virulence by influencing appressorial penetration and invasive growth [20,50,51]. In addition, fungi synthesize, fold, and mature a series of secreted proteins through the ER to influence the infection by affecting the host defense response during the biotrophic phase of pathogenic fungi infection of the host plants [52,53]. Similarly, Δ*Mocbp7* displayed obvious pathogenic defects, as not only can most appressoria still not form the penetration peg at 48 hpi but also have a defect in invasive hyphae (Figure 5D,E). These indicated that MoCbp7 is involved in some basal ER homeostatic mechanisms of *M. oryzae* including the UPR pathway, ER-phagy, and protein secretion, therefore affecting fungal development and pathogenicity. However, it is interesting to note that Crz1 is not involved in the ER stress response in fungi [14,54]. Deletion of MoCrz1 would make fungi sensitive to cell wall stress factors [9], but Δ*Mocbp7* was not sensitive to cell wall stress. Calcium transporters Yvc1 and Vcx1, located on the vesicle membrane, were expressed in a calcineurin/MoCbp7-dependent manner, but they were not downstream targets of *CRZ1* [47] (Figure 3C). Therefore, it is reasonable to believe that there are other alternative calcium signal pathways that exist in fungi in addition to the Crz1-involved calcium signal pathway, especially in the regulation of ER homeostasis, in which MoCbp7 is involved (Figure 6). Hence, a possible model was proposed to intuitively illustrate the functions of MoCbp7 in the calcineurin-mediated calcium signaling pathway as well as its potential connection with MoCrz1, in which MoCbp7 and MoCrz1 are both involved in the regulation of calcium ion homeostasis through calcineurin-mediated calcium signaling, which is crucial for fungal development and virulence in *M. oryzae* (Figure 7). In *M. oryzae*, appressorial penetration depends on turgor pressure and rapid actin polymerization. When turgor pressure reaches a critical threshold, septin GTPases assist in the re-orientation of actin to the base of the appressorium, converting the turgor pressure into a strong mechanical force and forming a penetration peg to breach the host cuticle [55]. Calcium signaling, as a conserved signaling pathway, is a participant in the reorganization of the actin cytoskeleton in a variety of organisms [56,57] and fungi hypha orientation [58], where transient calcium influx is often accompanied by the depolymerization of F-actin and, after calcium ion diffusion, by actin reaggregation [59]. In *M. oryzae*, local calcium flux can be measured to be induced in the developing appressorium [60]. The Δ*Mocbp7* mutant showed normal turgor pressure, but the appressorial penetration was severely defective (Figure 5), indicating that the defect of the Δ*Mocbp7* mutant virulence may be associated with a functional defect of the cytoskeleton, and MoCbp7 may also act as an important element involved in appressorium repolarization regulated by calcium signals.

In summary, we identified a novel calcineurin regulatory-subunit-binding protein, MoCbp7, which has an important role in the calcium signaling pathway. MoCbp7 is essential for growth, conidiation, appressorium formation, and pathogenicity. Our study provided a new perspective on the role of the calcium signaling pathway in *M. oryzae*, and further attempts can be made to develop more accurate calcium signaling monitoring tools for the calcium signaling study in *M. oryzae*.

## 4. Materials and Methods

### 4.1. Fungal Strains and Culture Conditions

*M. oryzae* 70-15 was used as the wild-type strain (WT) for this experiment, and all fungal strains were cultured in solid complete medium (CM) agar plates at 25 °C with a 16 h of light/8 h of darkness photoperiod for 8–10 days [61]. For different stress tests, 0.3 mM CaCl_2_ was added to solid CM agar plates, and 1 μg/mL tunicamycin (TUNI) and 5 mM dithiothreitol (DTT) were added to solid MM agar plates, respectively.

### 4.2. Generation and Complementation of the Null Mutant

The Δ*Mocbp7* mutant was obtained by a high-throughput knockout system that was slightly modified [62]. The upstream and downstream fragments (approximately 1500 bp) of *MoCBP7* were amplified from the genome of the wild-type 70-15, and a hygromycin-resistance gene (HPH) fragment (approximately 1300 bp) was cloned from the pCB1003 vector. The three fragments were fused into the *Xba*I/*Hin*dIII site of PKO3A using a fusion enzyme (Transgen, Beijing, China). Then, the gene-deletion cassette was introduced into the wild-type 70-15 by *Agrobacterium tumefaciens*-mediated transformation (ATMT) [63]. The copy number of HPH in the genome of the deletion mutant was verified with quantitative real-time PCR (Figure S1). The full-length genomic sequence of *MoCBP7*, including the native promoter, was amplified from the genomic DNA of wild-type strain 70-15 and then fused with the *Eco*RI*/Sma*l*-*linearized vector PKD5 using the fusion enzyme (Transgen, Beijing, China). The resulting recombinant vector was introduced into the mutant strain by the ATMT method. All the primers required for gene knockout and complementation are listed in Table S2.

### 4.3. Quantitative Real-Time PCR Analysis

Following the manufacturer’s instructions (TaKaRa, Kusatsu, Japan), total RNA was extracted from aerial mycelia grown for four days with TRIzol reagent. cDNA synthesis from RNA was achieved by the PrimeScript RT reagent Kit with gDNA Eraser (TaKaRa, Kusatsu, Japan). Quantitative real-time PCR, used for quantifying target gene expression levels, was performed on a real-time PCR detection system Mastercycler (Eppendorf, Hamburg, Germany) with the SYBR premix *Ex Taq* (Tli RNaseH Plus) kit (TaKaRa, Kusatsu, Japan). Four biological replicates were conducted for each experiment, with both *TUBULIN* and *H3* expression levels as controls. The method of data processing was carried out as previously described [20,64].

### 4.4. Protein Extraction and Western Blot Analysis

To detect the level of autophagy and endoplasmic reticulum autophagy, the GFP–MoAtg8 fusion protein or the GFP–MoSec62 fusion protein were transferred to the wild-type strain and Δ*Mocbp7* via the ATMT method. The strains were cultured in liquid CM medium for 40 h (25 °C, 150 rpm) then shifted to CM containing 5 mM DTT or tacrolimus (FK506) or were transferred to nitrogen-starved (SD-N) liquid medium. For the protein extraction, the harvested mycelia were collected and shattered in 500 uL of protein lysis buffer (500 mM Tris-HCl [pH 7.5], 150 mM NaCl, 1 mM EDTA, 1% Triton X-100) with 5 uL protease inhibitor (FDbio, Hangzhou, China). GFP-tagged fusion proteins and free GFP were probed with an anti-GFP antibody (HUABIO, Hangzhou, China). Densitometric analysis was performed on ImageJ (x64) 1.8.0 to quantify the degradation level of fusion proteins; GAPDH was used as a control.

### 4.5. Yeast Two-Hybrid Assays

The coding DNA sequences (CDS) of *MoCBP7* and *MoCNB1* were amplified and fused with a yeast two-hybrid prey expression vector, pGADT7 or pGBKT7. The primers were listed in Table S2. pGADT7-T and pGBKT7-53 were used as positive controls. pGBKT7-Lam and pGADT7-T were used as negative controls. According to the Matchmaker Gold Yeast Two-Hybrid System (Clontech, Mountain View, CA, USA), two recombinant plasmids were co-transformed into the yeast receptor cells and cultured in SD-Leu-Trp and SD-Leu-Trp-His-Ade at 30 °C.

### 4.6. Pull-Down Assays

The CDS region of *MoCBP7* was fused into the prokaryotic expression vector pET21a with a 3×FLAG tag, and the CDS region of *MoCNB1* was fused into the prokaryotic expression vector pGEX4T with a GST tag. The primers can be obtained in Table S2, and the fusion proteins were expressed in the *Escherichia coli* strain BL21 (DE3) using IPTG (isopropyl β-D-1-thiogalactopyranoside) as an inducer. After 16 h of induction, the cells were enriched and resuspended with binding buffer (50 mM Tris-HCl [pH 8.0], 150 mM NaCl, 10 mM Imidazole), followed by sonication to break up the cells. Protein supernatants containing GST–MoCnb1 and GST proteins were co-incubated with GST beads (BBI, Shanghai, China) for 2 h at 4 °C. After five washes with the wash buffer (BBI, Shanghai, China), protein supernatants containing 3 × FLAG–MoCbp7 protein were co-incubated with beads again for 2 h at 4 °C. The proteins were eluted using elution buffer (BBI, Shanghai, China) and detected by immunoblotting with an anti-GST antibody and an anti-FLAG antibody (HUABIO, Hangzhou, China).

### 4.7. Fluorescence Observation

The PKD5 vector fused with MoCbp7–GFP or the PKD8 vector fused with MoH2B-mCherry were co-transferred into the Δ*Mocbp7* mutant. For the subcellular localization analysis, conidia were harvested from 9-day-old CM agar plates and observed under a confocal fluorescence microscope (Zeiss LSM 710, 63 × oil, CarlZeiss, Jena, Germany). The conidia were induced on hydrophobic coverslips for appressorial formation and observed at 24 hpi. Mycelium was incubated in liquid CM for 24 h and observed.

### 4.8. Phenotype Assays

For the conidial germination and appressorium formation assay, 40 μL of 5 × 10^4^ conidia/mL conidial suspension was pipetted onto hydrophobic coverslips, placed at 22 °C in the dark, and observed under the microscope. For the strain virulence assays, the mycelial plugs were inoculated on the tips of isolated barley leaves and incubated at 25 °C with a 16 h of light/8 h of darkness photoperiod for 4 days. Additionally, the conidial suspension that was adjusted to a concentration of 5 × 10^4^ conidia/mL with 0.2% (*w*/*v*) gelatin was spray-inoculated on CO-39 seedlings, a susceptible host. The disease severity was assessed as previously reported [65,66]. For the appressorial penetration assays, conidial suspension at a concentration of 5 × 10^4^ conidia/mL was dropped on the intact and wounded barley leaves. All the leaves were decolorized by methanol, and the appressorium penetration was evaluated under the microscope.

## Data Availability

All data supporting the findings of the current study are available within figures and supporting information. All strains generated during this study are available from the corresponding author upon reasonable request.

## Supplementary Materials

The following supporting information can be downloaded at: https://www.mdpi.com/article/10.3390/ijms24119297/s1.
