# Supplementary material for: MoCbp7, a Novel Calcineurin B Subunit-Binding Protein, Is Involved in the Calcium Signaling Pathway and Regulates Fungal Development, Virulence, and ER Homeostasis in Magnaporthe oryzae
Source: Int J Mol Sci. 2023 May 26;24(11):9297. doi: 10.3390/ijms24119297 (PMC10252744; doi:10.3390/ijms24119297)
Supplement: Supplementary file 1 [file ijms-24-09297-s001.zip › ijms-2309498-supplementary.pdf]

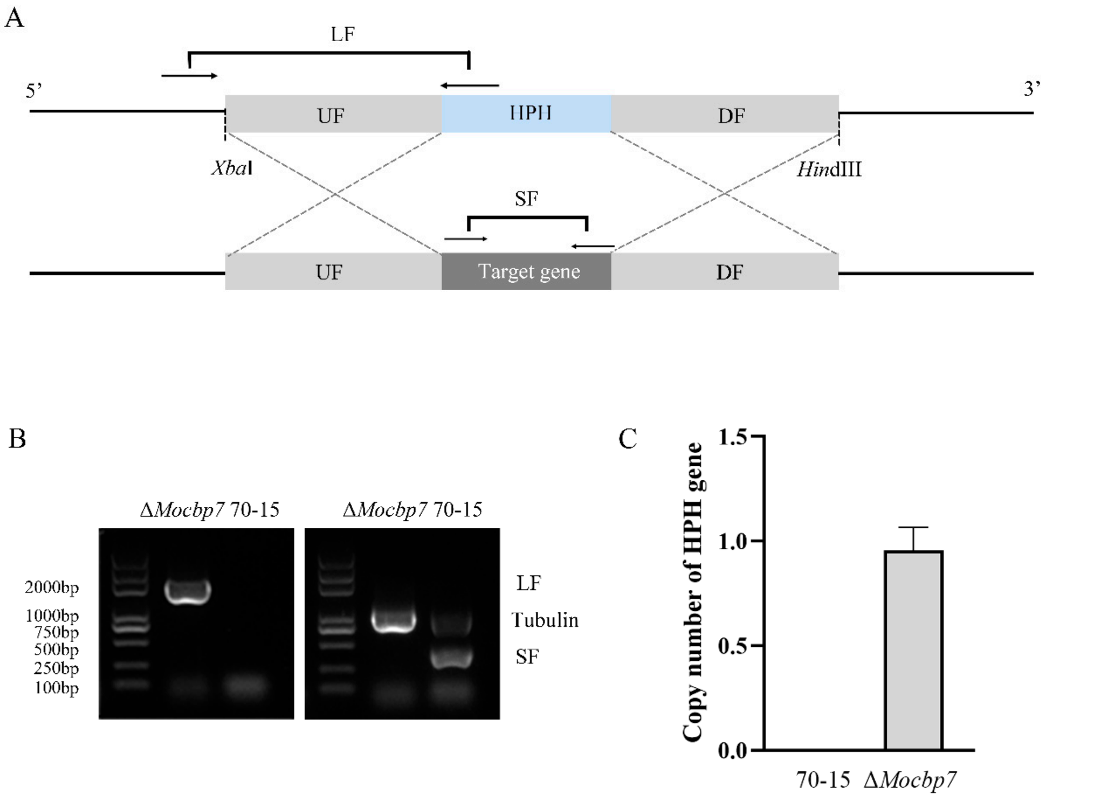

Figure S1. Identification of knockout mutants. (**A**) The knockout model in *M. oryzae*. (**B**) The Δ*Mocbp7* mutant was verified deletion of *MoCBP7* by PCR. (LF, long fragment; SF, short fragment) (**C**) Insertion copy number of the HPH gene in 70-15 and the Δ*Mocbp7* mutant.

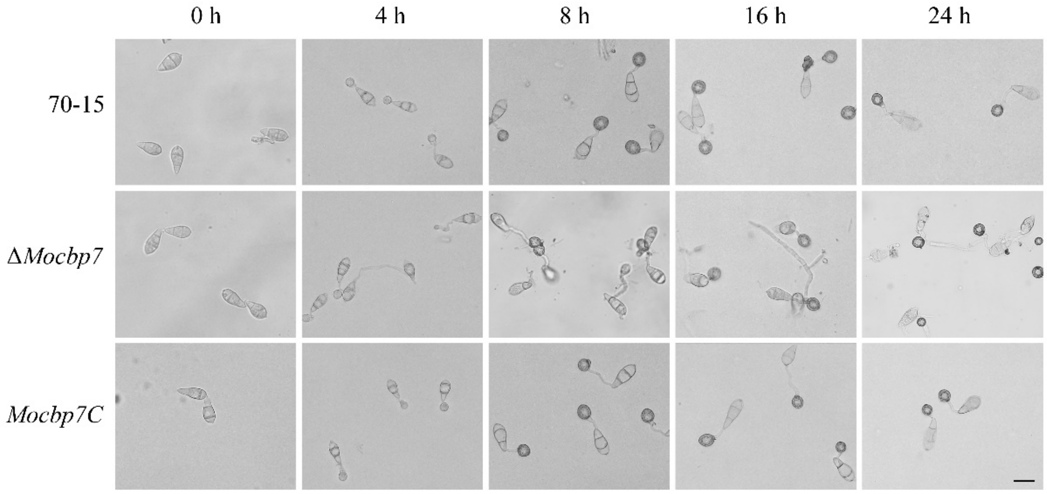

Figure S2. Appressorium formation images of conidia induced on hydrophobic coverslips at 0 hpi, 4 hpi, 8 hpi, 16 hpi, 24 hpi. Bar, 10 μm.

Table S1. List of potentially interacting proteins with MoCbp7 screened the rice blast fungus cDNA library.

**Gene ID**	**Gene description in NCBI**	**Validation of interactions by Y2H**
MGG_04764	cystathionine gamma-synthase	
MGG_10179	dolichyl-phosphate mannosyltransferase polypeptide 2	
MGG_06952	ribosomal protein L38e	
MGG_07467	pyruvate dehydrogenase kinase	√
MGG_10910	aflatoxin biosynthesis ketoreductase nor-1	
MGG_06044	ubiquitin-60S ribosomal protein L40	
MGG_08785	X-Pro dipeptidyl-peptidase	
MGG_03441	trehalose-phosphatase	
MGG_07218	transcription factor	√
MGG_14773	AGC/AKT protein kinase	
MGG_06728	meiotic recombination protein rec14	
MGG_15291	pheromone processing endoprotease KexB	
MGG_06936	elongation factor 1-gamma	
MGG_00703	MAS3 protein	
MGG_01742	elongation factor 2	
MGG_06933	calcineurin subunit B	√
MGG_03226	cytochrome b-c1 complex subunit 2	
MGG_04477	DNA-directed RNA polymerase III subunit	
MGG_02690	Arg-6 protein	
MGG_06505	mRNA cap guanine-N7 methyltransferase mRNA	
MGG_02634	coatomer subunit gamma	
MGG_10315	hydrophobin-like protein MPG1	
MGG_02086	proteasome component PRE3	
MGG_04978	E3 ubiquitin ligase complex SCF subunit scon-3	
MGG_08829	transcriptional repressor rco-1	
MGG_08832	C-5 sterol desaturase	
MGG_07110	phosphatidylethanolamine N-methyltransferase	
MGG_10447	peptidyl-prolyl cis-trans isomerase	√
MGG_04087	thiosulfate sulfurtransferase	
MGG_06860	coatomer subunit beta	
MGG_09310	dolichyl pyrophosphate	
MGG_07134	polyamine acetyltransferase	
MGG_01556	V-type proton ATPase subunit F	
MGG_05097	transmembrane domain-containing protein	
MGG_06152	GMF family protein	
MGG_05528	hob3	
MGG_01061	coproporphyrinogen III oxidase	

MGG_06722	1,3-beta-glucanosyltransferase gel2	
MGG_02953	60S ribosomal protein L21-B	
MGG_01135	cytochrome c oxidase polypeptide IV	
MGG_01282	polyubiquitin	
MGG_12122	CMGC/GSK	
MGG_09495	Arp2/3 complex subunit	√
MGG_06930	hypothetical protein	
MGG_06374	hypothetical protein	

Table S2. Primers used in this study.

**Primer name**	**Primer**
**Primers for gene knock out**	
CBP7-UPF	AGGCTAACTGACACTCTAGAGACGGCAAAGCCGGACAAAA
CBP7-UPR	TGTTGACCTCCACTACGCAAAGCACTTTCAGTCACG
CBP7-DOWNF	GGAATAGAGTAGATGCGCTTCCCATTTCACACG
CBP7-DOWNR	CGACGGCCAGTGCCAAGCTTTACCGCATGACAACATGCT
CBP7-LONG-F	GCGAGCTTAAGCACATCGAGC
CBP7-SF	CCAAATATCCTATCTCACGTC
CBP7-SR	AATGATATGGGGTTGTTGCTC
YHPH-R	GATAATAATGTCCTCGTTCC
HPH-F	TAGTGGAGGTCAACAATGAATG
HPH-R	CATCTACTCTATTCCTTTGCC
CBP7C-F	TGCCCGTCACCGAGATTTAG TGAGCAACATGGTACACGG
CBP7C-R	GTCGTGGTCCTTGTAGTC TGCAATGCTCCACACGGGCGC
Tubulin-F	ACAACTTCGTCTTCGGTCAG
Tubulin-R	GTGATCTGGAAACCCTGGAG
**Primers for quantitative real-time PCR**	
H3-F	CGAGCTTCTGATCCGCAAG
H3-R	TCAAAGAGCGAGACAAGGTATG
TUBULIN-F	GCAGATGTTCGACCCCAAG
TUBULIB-R	ACGAGTTCTTGTTCTGGACG
YVC-F	TGGTTCTCGCATCAATCCTC
YVC-R	CTTGTGCTGGAGATACTCTGTG
VCX-F	AGGACTCATTAACGCCACG
VCX-R	TCGATCTGCCCTTGTTTGAG
PMC1-F	GTTCGTGTGGATGCAAATTTTC
PMC1-R	TTGGTGATAAGTGCCTGAGC
PMR1-F	ACAAGTTACCAAGCTGAGGG
PMR1-R	GCAAGGACGAAGGACACAG
CMD1-F	CCTCGTTGATCATATCCTGCAG
CMD1-R	CCTCGTTGATCATATCCTGCAG
CNA-F	GTTTCTTTGTATTCACGGTGGC

CNA-R	AAGGATATCGCACATCAGGC
CNB-F	AAAGCTCCAACTTCGATAGGG
CNB-R	GCAATCATTCGTGTGGCAAG
RCN1-F	GCAAAACGATATCAGGCCATC
RCN1-R	TTGGTGAAGAGTAGGGTGTTG
CMK1-F	GAGCTTTTCGACCGTATTTGC
CMK1-R	GCTCAGGTAAAGTAGGTTCTCG
DUN1-F	GTCCTATGCCTGACACGAAC
DUN1-R	TGAGGCAGTGTCGATTTGAG
CRZ1-F	TGAGAAACTTTGAGGGCAGAC
CRZ1-R	CGGTACTTCCATTGGCATAAGG
SCJ1-F	GGTCACGTAGAGATGGTCAA
SCJ1-R	CCTGTATTTCTCCCACACTG
SIL1-F	GACTTAGAGGGTCTGCCTGT
SIL1-R	GAAGTACATGTCGTGGGAAA
ERV29-F	CGGCTACGGTCTTATTTTCG
ERV29-R	CAAAGCCGATGAAGAGGAAG
SEC61-F	AGCCATCTCCGGTCTTACCT
SEC61-R	CTGGTCCTTAAGCTGCTTGG
PMT4-F	GTTGGTGTTTTCGCCTTCAT
PMT4-R	CGTGAGGACTGCAAAGTGAA
PDI1-F	GTTGTCGCTTACTTGTCCAG
PDI1-R	CGTCAAATTTCTTGTCGAAG
WBP1-F	GAGGCCATTCCTCACCTACA
WBP1-R	TTTGGTAACGGTGGCTTTTC
PMT1-F	GGCGAGAAGAAGGACATCAA
PMT1-R	CCCTTGTCGTCTCGGTACTC
PPS1-F	CTTCCGAGATTGCACCAAAT
PPS1-R	AGTTGGTGGCAGAATTGACC
PMT2-F	GAGTCATTGGCCTGGTTGTT
PMT2-R	GAACGTCAGGATGGCAAAGT
KAR2-F	AACGGTCTCGAGAACTATGC
KAR2-R	TCCTTCTGCTCCTCAAAATC
RT-HPH-F	ATGTCCTGCGGGTAAATAGC
RT-HPH-R	GATGCAATAGGTCAGGCTCTC
RT-Tubulin-F	ACAACTTCGTCTTCGGTCAG
RT-Tubulin-R	GTGATCTGGAAACCCTGGAG
**Primers for yeast two hybrid**	
ATG16-AD-F	GGAGGCCAGTGAATTCATGTCTTCGCTGCCGGAC
ATG16-AD-R	CGAGCTCGATGGATCCTCCTCGTTTGGCGAATTTC
CNB1-AD-F	GGAGGCCAGTGAATTCATGGGCAACACGACCAGCAATG
CNB1-AD-R	CGAGCTCGATGGATCCGAATTGGTCTAGCGTCATGC
CBP7-BD-F	CATGGAGGCCGAATTCATGCCGTTCGGGCGCGCAG

CBP7-BD-R	GCAGGTCGACGGATCCTGCAATGCTCCACACGGG
**Primers for Pulldown**	
CBP7-FLAG-F	GACAAGGTCGACAAGCTTATGCCGTTCGGGCGCGCAGGC
CBP7-FLAG-R	GAGTGCGGCCGCAAGCTTTGCAATGCTCCACACGGGCGCC
CNB1-GST-F	CGTGGATCCCCGGAATTCATGGGCAACACGACCAGCAATG
CNB1-GST-R	GAGTCGACCCGGGAATTCGAATTGGTCTAGCGTCATGC
**Primers for fluorescence observation**	
CNB1-GFP-F	ATCACAATGGCCGGATCCATGGGCAACACGACCAGCAA
CNB1-GFP-R	CTTGCTCACCATCCCGGGGAATTGGTCTGCAAATCGT
H_2B-mCherry-F	ATGGTCGGATCCATCCCCGGGATGCCCCCCAAGGCCGCTGACAA
H_2B-mCherry-R	TTACTGCAGGTCGACTCTAGAGCCGCCGGTGGAGTGGCGGCC
